# Atypical Flexibility in Dynamic Functional Connectivity Quantifies the Severity in Autism Spectrum Disorder

**DOI:** 10.3389/fnhum.2019.00006

**Published:** 2019-02-01

**Authors:** Vatika Harlalka, Raju S. Bapi, P. K. Vinod, Dipanjan Roy

**Affiliations:** ^1^Center for Computational Natural Sciences and Bioinformatics, IIIT Hyderabad, Hyderabad, India; ^2^Cognitive Science Lab, IIIT Hyderabad, Hyderabad, India; ^3^School of Computer and Information Sciences, University of Hyderabad, Hyderabad, India; ^4^Cognitive Brain Dynamics Lab, National Brain Research Centre, Manesar, India

**Keywords:** resting-state functional MRI, autism, flexibility, dynamic connectivity, ABIDE

## Abstract

Resting-state functional connectivity (FC) analyses have shown atypical connectivity in autism spectrum disorder (ASD) as compared to typically developing (TD). However, this view emerges from investigating static FC overlooking the whole brain transient connectivity patterns. In our study, we investigated how age and disease influence the dynamic changes in functional connectivity of TD and ASD. We used resting-state functional magnetic resonance imaging (rs-fMRI) data stratified into three cohorts: children (7–11 years), adolescents (12–17 years), and adults (18+ years) for the analysis. The dynamic variability in the connection strength and the modular organization in terms of measures such as flexiblity, cohesion strength, and disjointness were explored for each subject to characterize the differences between ASD and TD. In ASD, we observed significantly higher inter-subject dynamic variability in connection strength as compared to TD. This hyper-variability relates to the symptom severity in ASD. We also found that whole-brain flexibility correlates with static modularity only in TD. Further, we observed a core-periphery organization in the resting-state, with Sensorimotor and Visual regions in the rigid core; and DMN and attention areas in the flexible periphery. TD also develops a more cohesive organization of sensorimotor areas. However, in ASD we found a strong positive correlation of symptom severity with flexibility of rigid areas and with disjointness of sensorimotor areas. The regions of the brain showing high predictive power of symptom severity were distributed across the cortex, with stronger bearings in the frontal, motor, and occipital cortices. Our study demonstrates that the dynamic framework best characterizes the variability in ASD.

## Introduction

Autism spectrum disorder (ASD) is a neuro-developmental disorder encompassing a range of disorders including Asperger's syndrome and pervasive developmental disorder. Resting state functional MRI, which measures blood oxygen level-dependant signals (BOLD) (Deco et al., 2013; Lee et al., 2013) has been used to study both physiology and pathology. The statistical analysis of the matrix obtained by using Pearson cross-correlation of the regional BOLD time series across pairs of regions of interest (ROIs) has been found to reveal several properties that are of clinical relevance. Recently, a number of studies have explored brain networks as graphs (van den Heuvel and Hulshoff Pol, 2010; Sporns, 2013). Graph-theoretic analyses have shown that the functional connectivity in the human brain is divided into well-organized modules or subnetworks which are densely connected within themselves and sparsely connected to each other. Disease and age affect this modular organization (Chen et al., 2013; Song et al., 2014; Ye et al., 2015). Song et al. (2014) reported that modularity decreases with aging, suggesting less distinct functional divisions and specialization across whole brain networks. They reported a decline in cognitive functioning and intact primary information processing with age in typical development (TD).

Several studies on neurocognitive diseases have reported atypical fluctuation in modularity. Recently, in ASD, alterations in the network properties (global and local efficiency, assortativity, clustering coefficients, characteristic path length, small world properties, etc.) with maturation and disease were found in our and other studies (Rudie et al., 2013; Harlalka et al., 2018b; Henry et al., 2018). Using static networks, a significant decrease in modularity has been observed. Both the functional connectivity *between* major networks (i.e., functional segregation) and connectivity *within* different networks (i.e., functional integration) are altered in ASD (Rudie et al., 2012, 2013). Studies have shown under-connectivity in various functional networks, especially in the Default Mode Network (DMN) (Hahamy et al., 2015; Yerys et al., 2015).

However, in recent times, it has been found that analyses based on static functional connectivity have several shortcomings. Static functional connectivity (FC) does not sufficiently incorporate time-varying (or dynamic) changes that occur through the brain scan (Chang and Glover, 2010). Dynamic functional connectivity (dFC) analyses have shown to reveal patterns of brain states that occur commonly as well as transitions among them (Damaraju et al., 2014; Preti et al., 2017). They also give an idea about the dynamic reconfiguration that occurs during tasks (Bassett et al., 2011; Braun et al., 2016; Gerraty et al., 2018). To analyze dynamic or instantaneous connectivity, the BOLD timeseries is divided into overlapping intervals, and a functional correlation matrix is derived for each of these intervals. The sliding window or tapered sliding window approach has been widely used to study dynamic connectivity of functional brain networks estimated from fMRI BOLD data (Xu and Lindquist, 2015; Park et al., 2017). With recent studies reporting that modularity of dynamic functional networks varies on very short timescales (Betzel et al., 2016, 2017), there is a possibility of tracking instantaneous changes in functional connectivity between brain regions. Particularly, while static communities represent sub-networks densely connected among themselves and sparsely connected to the rest of the brain, community structures in dynamic networks would project the ongoing changes in communities over time (Bassett et al., 2011; Cole et al., 2013).

Recent studies have explored the dynamic nature of atypical information processing in ASD (Falahpour et al., 2016; Chen et al., 2017; de Lacy et al., 2017; Watanabe and Rees, 2017; Rashid et al., 2018). Watanabe and Rees (2017) reported that the autistic brain shows fewer state transitions compared to those of typically developing, and such atypically stable brain dynamics relates to symptom severity in ASD. Some studies reported altered dynamic functional connectivity between specific areas (Falahpour et al., 2016). Several whole-brain studies found dominant brain states as well as their dwell times. Resting state fMRI analysis of ASD cohort reveals that the dynamics spends more time in globally disconnected states with fewer state transitions as compared to TD (de Lacy et al., 2017; Rashid et al., 2018). Flexibility of brain also provides an alternate way of quantifying the dynamic changes in fMRI studies (Garcia et al., 2018). Intuitively, it can be thought of as a measure to quantify the dynamic reconfiguration that occurs in the brain over time. It has been applied to obtain insights into altered dynamic pattern in various disease states such as schizophrenia (Braun et al., 2016) and epilepsy (Tailby et al., 2018). The influence of resting-state flexibility on disease and age has not been studied, particularly in ASD.

**Figure 1:**
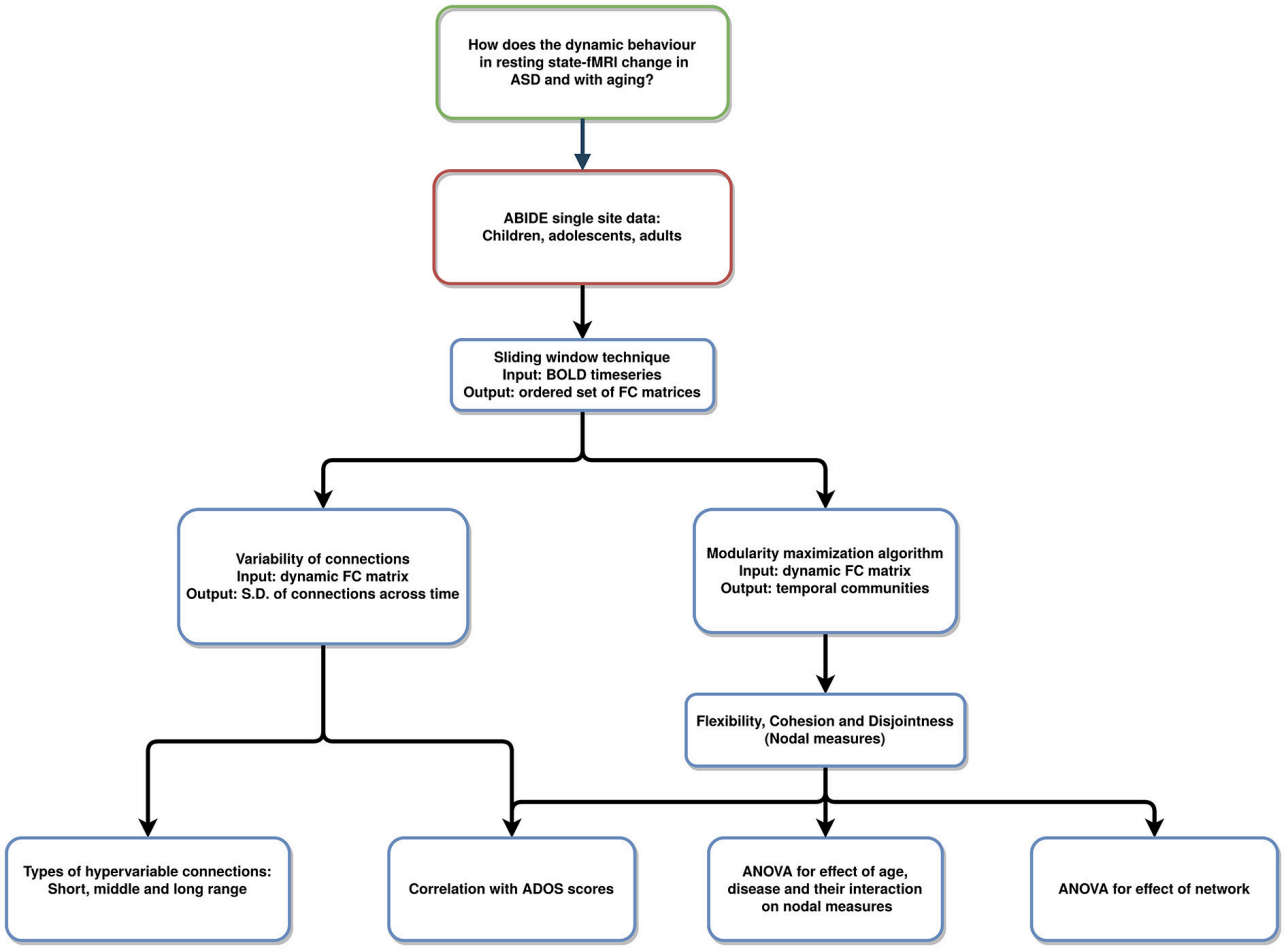
Pipeline of the analysis: resting-state fMRI data used to analyze varying temporal dynamics in ASD and TD.

Therefore, we adopt this framework to investigate the dynamic changes in functional activity of TD and ASD. We stratified the data (ASD and TD) into groups of children, adolescents, and adults. We attempted to quantify the variability in dynamic FC in two plausible ways. Firstly, we quantified the variability in the connection strength. Secondly, dynamic metrics, flexibility, cohesion, and disjointness were used to study the differences between ASD and TD. The pipeline is summarized in Figure 1.

## Materials and Methods

### Data Acquisition and Preprocessing

We used single-site data (NYU) from the ABIDE Preprocessed Initiative (Cameron et al., 2013). Institutional review board approval was provided by each data contributor in the ABIDE database. Detailed recruitment and assessment protocols and inclusion criteria are all available on the ABIDE website. The data was preprocessed using the DPARSF pipeline (Data Preprocessing Assistant for Resting State fMRI) (Chao-Gan and Yu-Feng, 2010). Briefly, acquisition time correction and head motion correction (Friston 24-parameter model) were done for each subject. The functional images were re-aligned using a 6 degrees-of-freedom transformation. The structural image was co-registered with the mean functional image. The following nuisance signals were removed as part of the ABIDE preprocessing initiative: head motion effects (using Friston 24-parameter model), signals from white matter and cerebrospinal fluid, linear, and quadratic trends. The images were warped into MNI space. Temporal filtering (0.01–0.1 Hz) was performed on the regressed time series (Liu and Duyn, 2013). Global signal regression (GSR) has been shown to cause anti-correlation in resting state brain networks and to distort group differences in intrinsic functional connectivity (Murphy et al., 2009; Weissenbacher et al., 2009; Saad et al., 2012). Therefore, we did not use GSR. Details of the preprocessing steps can be found here: http://preprocessed-connectomes-project.org/abide/dparsf.html. We used the Automated Anatomical Labeling template (AAL) for parcellating the brain into 90 regions of interest (ROIs). We have provided the mapping between names of the ROIs and their abbreviations in Supplementary Table 1.

ABIDE Preprocessed Initiative (Cameron et al., 2013) provides visual assessment of the functional data from 3 manual functional raters. Subjects were excluded if (1) Any of the 3 raters gave it a “Maybe” or a “Fail” rating. (2) failed visual inspection of anatomical images and surfaces; (3) mean framewise displacement > 0.1 mm. Further, the data was age-stratified into three cohorts: young children under 11 years of age, adolescents from 11 to 18 years of age, and adults above 18 years of age (Table 1). Overall, we did not find significant differences in within-group properties for age and IQ between ASD and TD subjects. Non-parametric *t*-tests were used to calculate differences in mean relative motion and IQ scores. While motion was slightly higher in children compared to adolescents and adults, we found that there were no significant differences in these measures between ASD and TD.

**Table 1:** Demographic details of the samples included in this study.

	**ASD**	**TD**	***p***
**CHILDREN**			
Age: mean (SD)	9.51 (1.12)	9.10 (1.32)	0.241
range	7.15–10.06	6.47–10.86	
Gender	24M/2F	19M/7F	
FIQ	76–142	80–136	0.103
ADOS total score (SD)	10.89 (4.2)	–	
mean FD (SD)	0.08 (0.04)	0.05 (0.02)	0.091
**ADOLESCENT**			
Age: mean (SD)	13.71 (1.79)	14.01 (1.74)	0.362
range	11.01–17.88	11.32–16.93	
Gender	23M/5F	23M/5F	
FIQ	78–132	80–121	0.526
ADOS total score (SD)	11.45 (4.46)	–	
mean FD (SD)	0.07 (0.03)	0.06 (0.03)	0.303
**ADULT**			
Age: mean (SD)	24.13 (3.92)	25.41 (5.87)	0.325
range	18.58–39.1	18.59–31.78	
Gender	14M/4F	14M/4F	
FIQ	80–137	81–139	0.607
ADOS total score (SD)	10.82 (3.9)	–	
mean FD (SD)	0.05 (0.03)	0.05 (0.02)	0.202

### Dynamic FC

We used the DynamicBC toolbox (Liao et al., 2014) for Dynamic FC creation. We used a tapered sliding window length of 30 s in accordance with previous studies (Allen et al., 2014; Betzel et al., 2016) and the window was moved with a stride of 1. A set of sliding window correlation matrices was calculated for each subject. A Fisher Z-Transformation (to transform the Pearson's *r*, i.e., the correlation coefficient) was then applied to improve the normality of the distribution of the correlation matrices. Finally, the dynamic FC variability matrix, dFCvar was calculated for each subject where *D*(*i*,*j*) is the standard deviation of the connection strength between ROIs *i* and *j* across the temporal windows. This matrix has also been referred to as the *connection flexibility matrix* in previous studies (Bassett et al., 2011; Betzel et al., 2016). Therefore, a higher *D*(*i*,*j*) would imply a hyper-variable connection between areas *i* and *j*. Then the Network Based Statistics (NBS) method (Zalesky et al., 2010) was used to find a significantly different component (*p*_connection < 0.01, *p*-cluster < 0.05, 10,000 iterations), both hyper-or-hypo-variant in the dFCvar matrix between ASD and TD in each age group. The significant variable connections are classified into short, middle, and long range connections. All the connections i.e., distances between the centers of every two ROIs in the AAL atlas were listed and sorted. The shortest 33% connections are *short-range*, highest 33% connections are *long-range* and the other connections are defined as *intermediate-range*.

We also estimated the correlation between dFCvar matrix and severity score of ASD. We used the permutation method for calculating significance of correlation with null hypothesis of no correlation between each edge and the ADOS scores (FDR corrected). More details about the methods used for network correlation with ADOS score as well as supplementary analysis to check for robustness of our results with different window parameters are provided in the Supplementary Information.

### Modularity Maximization

We used the modularity maximization algorithm to determine the temporal communities in the multilayer dynamic functional connectivity matrix. For visualization, one can imagine a super adjacency matrix consisting of multiple adjacency matrices. Multilayer modularity algorithm finds communities within this super adjacency matrix. Similar to static networks, regions in the same temporal communities are also expected to have higher intra-community connectivity compared to regions in different communities. This algorithm identifies communities across time by maximizing a Louvain-like modularity function (Q):

$$\begin{array}{l} Q=\frac{1}{2\mu }\underset{ijlr}{\sum }\left(\left(A_{ijl}-\gamma_{l}.P_{ijl}\right)\delta_{lr}+\delta_{ij}.\omega_{jlr}\right)\delta \left(g_{il},g_{jr}\right) \end{array}$$

In the above expression, *A*_*ijl*_ is the correlation of regions *i* and *j* in layer *l*. *P*_*ijl*_ is the expected correlation in an appropriate null model. Two free parameters γ and ω, are used to scale the number of communities and the strength of the inter-layer edges that link each node to itself, respectively. In our study, we used the Newman-Girvan null model and the default values of 1 for the two free parameters. We used the Genlouvain Matlab toolbox (Jutla et al., 2011–2012) to calculate the community assignments. Since the output could vary in each run due to the stochastic nature of optimizing the partition function, we repeated the algorithm 50 times and calculated the three dynamic metrics—flexibility, cohesion, and disjointness for each run. The final value of each metric for each subject is the average of the 50 runs.

### Flexibility

The multilayer modularity maximization algorithm detected the community affiliations of each ROI at each window i.e., the output is *G* = *N* × *T* matrix where each element *(n,t)* is the community that ROI *n* belongs to at window *t*. Regional flexibility (*f*) of an ROI *i*, is defined as the ratio of the number of times it changed its community affiliation through the temporal windows to the possible number of community changes (as shown in the expression below). It is a number between 0 and 1 where zero implies most rigidity and one implies the most flexibility in terms of community changes through time.

$$\begin{array}{l} f_{i}=1-\frac{1}{T-1}\overset{T-1}{\underset{s=1}{\sum }}\delta \left(G_{i,s},G_{i,s+1}\right) \end{array}$$

Nodes with low flexibility form the rigid temporal core while nodes with high flexibility form the temporal periphery. Global flexibility (F) for a subject is calculated as the mean flexibility score across all ROIs ($F=\frac{1}{N}\overset{N}{\underset{i=1}{\sum }}f_{i}$). For our analysis, we calculated both the regional and global flexibility scores for 50 runs of the modularity algorithm and averaged the values across runs for statistical analysis. We also correlated the regional and global flexibility scores with the subject-wise ADOS scores and the modularity of each subject. Further, we repeated the analysis with different values for parameters γ and ω of the multilayer modularity algorithm.

### Relationship Between Modularity and Flexibility

Static modularity is a metric that quantifies the subdivision of a network into communities. Higher modularity implies higher within-module connectivity and lower between-module connectivity. We first calculated the static FC using the pearson cross-correlation of the BOLD timeseries. We binarized the matrix using 10% proportional thresholding. We used the Louvain algorithm (Blondel et al., 2008) to calculate the modularity for each subject. Non-parametric correlation method was used to calculate the correlation between global/regional flexibility scores of the ROIs and modularity.

### Cohesion and Disjointness

Although flexibility characterizes the modular changes in each brain area through the scan time, it does not capture changes in community affiliation. For this, we use two network measures: Cohesion strength and node disjointness. These are complementary measures of dynamic networks. Node disjointness quantifies the fraction of times a node changes its community independently, without other nodes from its module. Node cohesion strength is calculated as a cohesion matrix, where the edge weight denotes the number of times a pair of nodes changes to the same community together. Cohesion strength of an ROI *i* is the sum of its row values in the cohesion matrix. We analyzed the correlations between ADOS scores and cohesion/disjointness.

## Result

### Hyper-Variance of Connections in ASD

**Figure 2:**
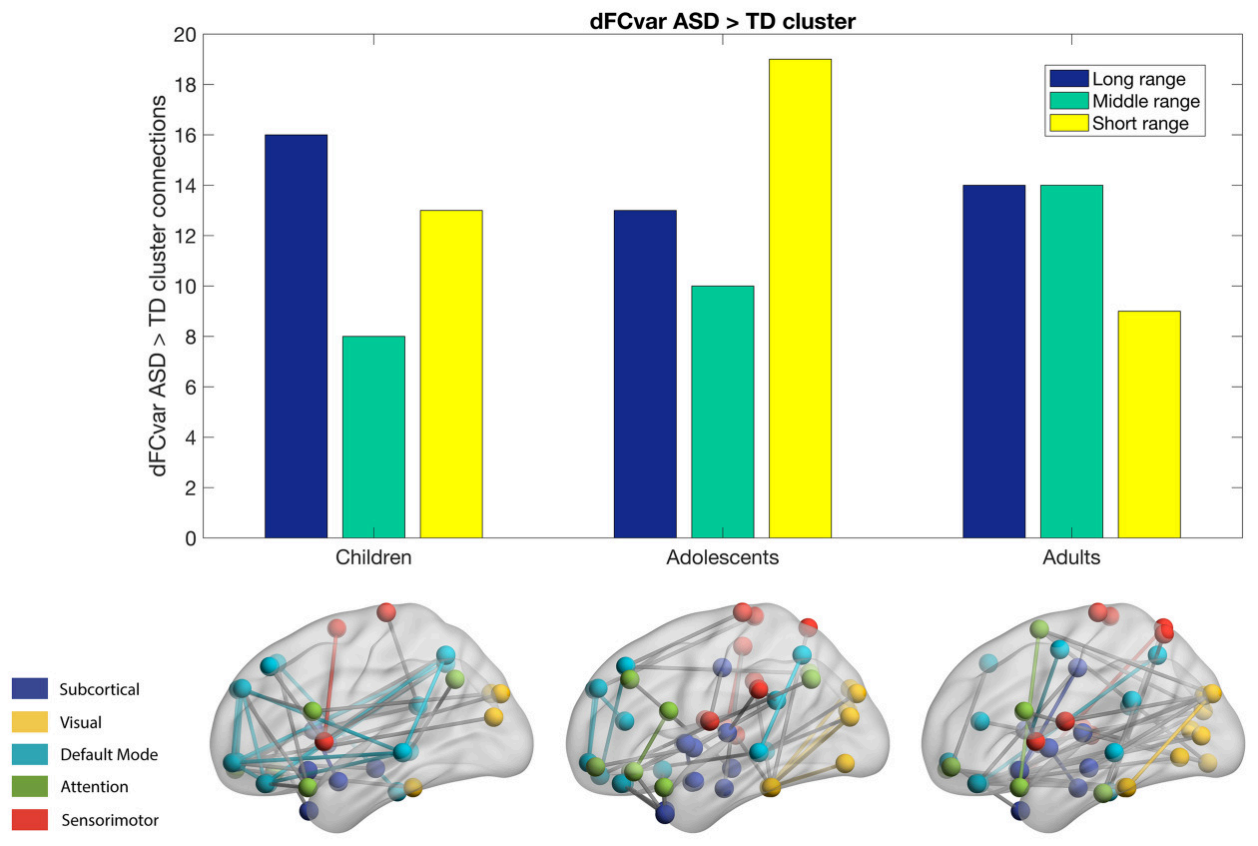
Hypervariant ASD connections in the dFCvar matrix. Majority of connections in children and adults are long-range while adolescents are seen to have majority short-range connections.

We calculated the dFCvar matrix, for every subject where each connection *(i,j)* of dFCvar is the standard deviation of the connection strength between ROIs *i* and *j* across the temporal windows. We found significantly hypervariant cluster of connections in the ASD group in all age groups—children, adolescents and adults. In the children group, the cluster had 37 connections. Most of these were intra-modular DMN-DMN connections, and most were long-range connections (43%). In the adolescents group, the cluster had 42 connections, around one third of which were intramodular and most were short-range connections (45%). Interestingly, in adults, similar to children, we observed high number of long-range (37%) and medium-range (37%) connections (Figure 2).

### Relationship Between dFCvar and ADOS Scores

We found that there were 8 hypervariant connections that showed very significant correlation (*r* > 0.4, *p* < 10^−5^, survived FDR correction) with ADOS score (Table 2). These connections did not show effect of age (one-way ANOVA, *p* > 0.05) indicating that this correlation was present in all age groups. These mostly included connections between inferior parietal areas and temporal regions. At the network level, we found that the mean DMN dFCvar connections showed significant correlation (*r* = 0.34, *p* < 0.05) with ADOS score as well as the mean DMN-Attention dFCvar connections (*r* = 0.39, *p* < 0.05). These too, did not show age effect with one-way ANOVA indicating that this effect is present for all age groups. However, the average functional connectivity did not show significant correlation with ADOS total scores (*p*-values did not survive permutation testing). All within-network and between-network correlations are reported in Supplementary Table 2. Overall, this shows that the inter-subject connection variability relates to symptom severity.

**Table 2:** Connections showing significant correlation (*p* < 10^−5^) to ADOS total score.

**ROI_1**	**ROI_2**	**Correlation**
Frontal_Inf_Orb_R	Parietal_Inf_L	0.47
Parietal_Inf_L	Temporal_Mid_L	0.42
Parietal_Inf_L	Temporal_Inf_L	0.45
Precentral_L	Insula_R	0.43
Precentral_L	Putamen_L	0.48
Supp_Motor_Area_R	Insula_R	0.44
Insula_L	Precuneus_L	0.47
Caudate_R	Temporal_Pole_Mid_R	0.43

### Negative Correlation of Flexibility and Modularity

**Figure 3:**
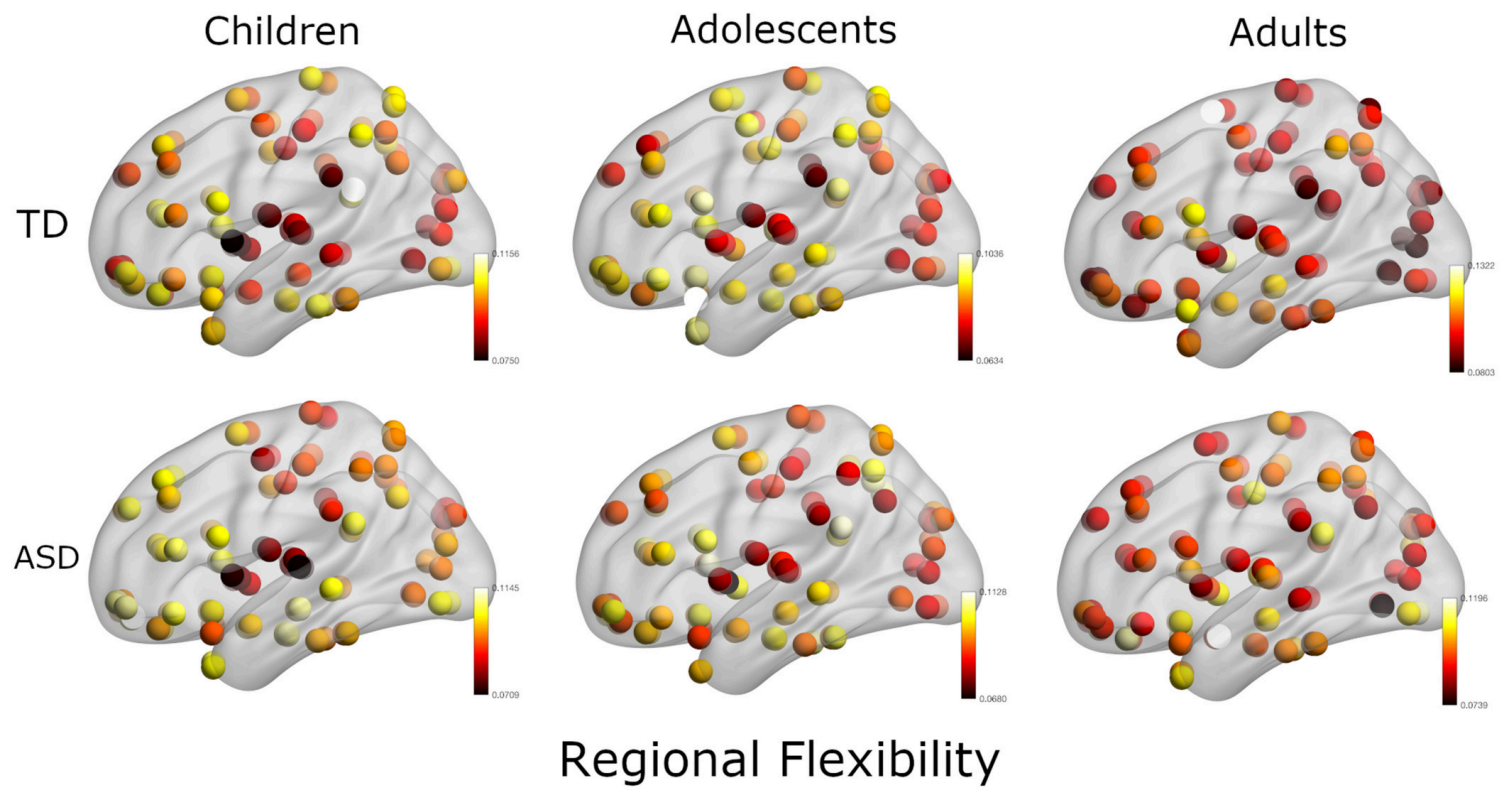
Plot of regional flexibility values for TD and ASD groups where the data is stratified into three groups: Children, adolescents, and adults.

In the next analysis, we calculated the multilayer dynamic FC using a multilayer modularity algorithm to partition the brain regions into communities across layers. We found that each subject had between 8 and 26 distinct communities (mean: 18.1). The flexibility of each region is calculated as the fraction of times the region changed its community assignment (Figure 3).

**Figure 4:**
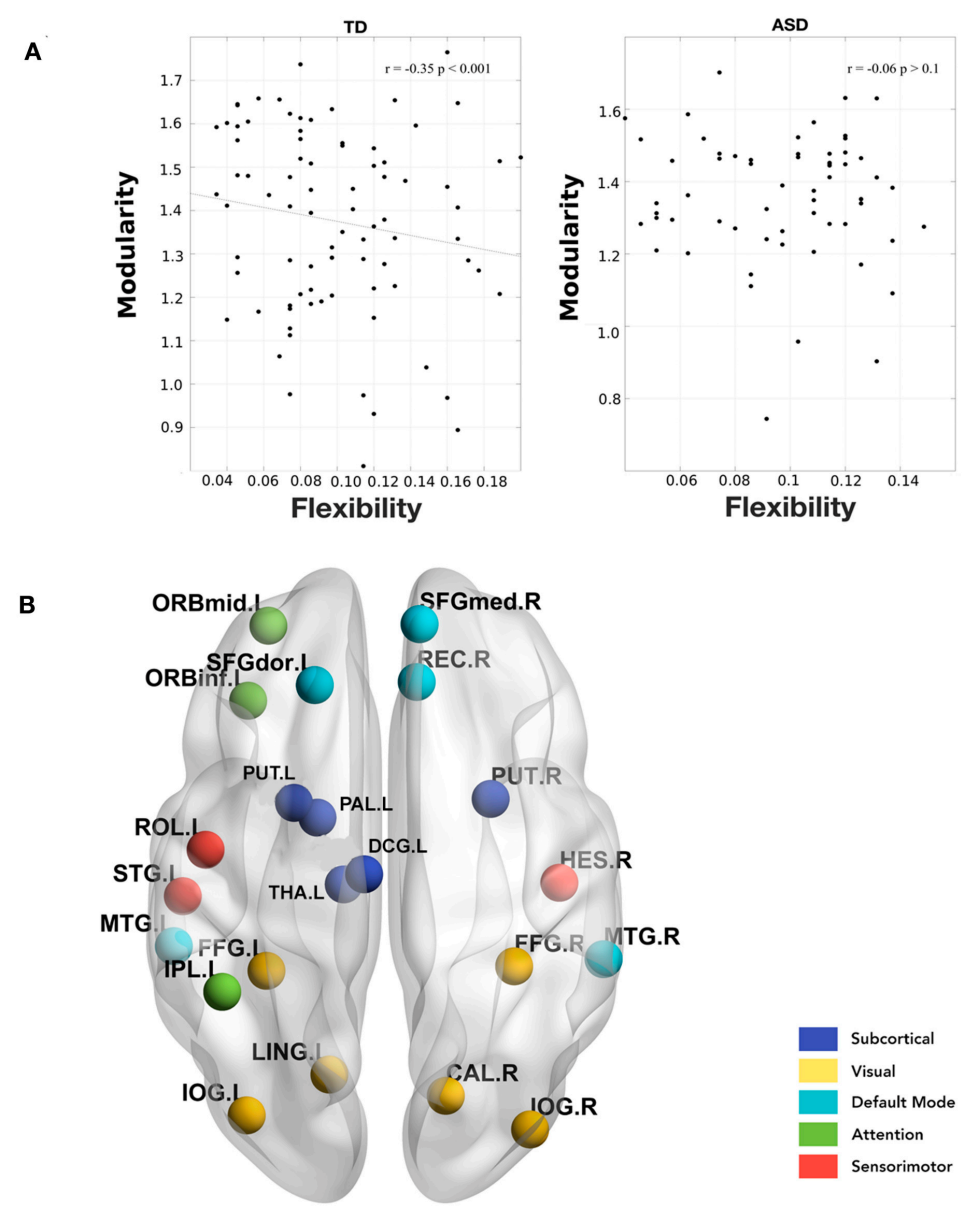
**(A)** Correlation between whole-brain mean flexibility score and modularity. A significant weak negative correlation is observed only in TD group. **(B)** Areas showing a significant negative correlation between local flexibility score and modularity in TD adults. None of these are significant in the ASD group.

Further, we calculated the modularity of static functional connectivity for each subject using Louvain algorithm and correlated it with the local and global flexibility values. In dynamic systems, different brain connectivity configurations can be interpreted as different attractor states. While modularity measures the depth of the attractor states, flexibility measures the frequency of the brain transitioning between states. Deeper states are more stable and will be more resistant to change, and so the regions in these states will have lower flexibility and vice versa (Ramos-Nuñez et al., 2017). We found that in the TD group, the mean whole-brain flexibility shows a significant (*p* = 0.001) correlation (*r* = −0.35) with modularity (Figure 4A). However, ASD did not show a correlation between flexibility and modularity. On stratifying the data based on age group and then calculating the correlation, we found that in the children's age group, TD did not show any significant correlation of whole brain mean flexibility with modularity score (TD: *r* = −0.06, *p* > 0.1, ASD: *r* = −0.08, *p* > 0.1). In adolescents and adults, we found that TD showed a significant correlation (*r* > −0.39, *p* < 0.01).

Further, we found that there were several areas that showed a significant correlation between local flexibility score and modularity in TD while only two of these areas (rectus gyrus and paracentral lobule) showed this correlation in the ASD group (Figure 4B). The areas showing significant correlation in TD group include DMN areas: middle temporal gyrus (MTG), gyrus rectus (REC), superior frontal medial, and dorsal gyrus (SFGdor, SFGmed); attention areas: inferior and middle frontal orbital areas (ORBinf, ORBmid) and inferior parietal areas (IPL); subcortical areas: putamen (PUT), pallidum (PAL), thalamus (THA); visual areas: calcarine fissure (CAL), occipital inferior gyrus (IOG), and fusiform gyrus (FFG); sensorimotor/auditory areas: rolandic operculum(ROL), heschl gyrus (HES), superior temporal yrus (STG), supplementary motor area (SMA).

### Significant Network Level Differences in Dynamic Network Measures

**Figure 5:**
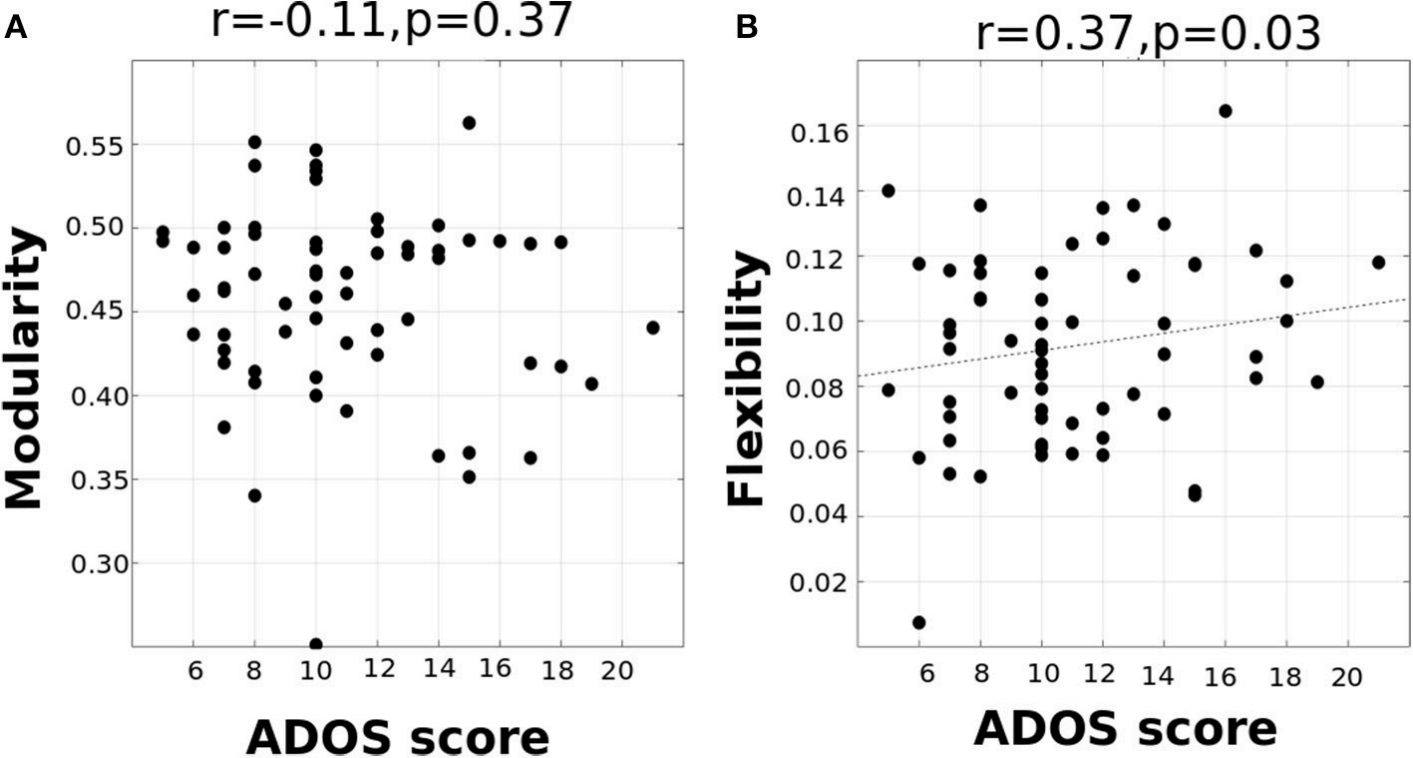
**(A)** correlation between ADOS total score and modularity **(B)** correlation between ADOS score and visual/auditory system flexibility (*p* < 0.05).

To examine if changes in whole-brain flexibility are driven by a biologically relevant organization of brain regions or instead driven by randomness/noise in the whole brain, we tested the average flexibility of functional networks (DMN, Attention, Subcortical, Sensorimotor, and Visual networks). We conducted a repeated-measures ANOVA with functional network as a within-subject factor and with age and disease as between-subject factors. We found a significant main effect of functional network [*F*_(4, 142)_ = 3.8, *p* < 0.0001], while there was no significant effect of interaction of network and disease [*F*_(4, 142)_ = 0.84, *p* = 0.13], or interaction of network, disease and age [*F*_(8, 142)_ = 1.61, *p* = 0.16]. The significant differences are listed in Supplementary Table 3. Overall, the results primarily indicate that the visual and sensorimotor areas show the least flexibility and the highest standard deviation indicating that they form the rigid temporal core. This is in contrast to DMN, Subcortical and Attention areas which have higher flexibility and lower standard deviation that form the flexible temporal periphery (See Supplementary Tables 3, 4). Although there is no disease effect observed at the network-level, it is possible that variation in ASD is not captured when it is considered as one group in the ANOVA analysis. Interestingly, we found that at the network level, mean visual, and auditory flexibility scores show a positive correlation with the ADOS scores. However, static modularity scores did not correlate with the ADOS scores (Figures 5A,B).

To further explore the differences in the dynamics at the network level, we also analyzed other dynamic measures: cohesion strength and disjointness. We observed that cohesion strength shows a significant network level effect [*F*_(4, 142)_ = 3.9, *p* < 0.01] with sensorimotor network showing high cohesion strength. There is a significant effect of network [*F*_(4, 142)_ = 43.05, *p* < 0.0001] on node disjointness with DMN network having highest disjointness and sensorimotor, visual networks having lowest disjointness. This analysis provides insights into the organization of the dynamic resting state functional brain.

### Effect of Age and Disease on Dynamic Metrics

**Figure 6:**
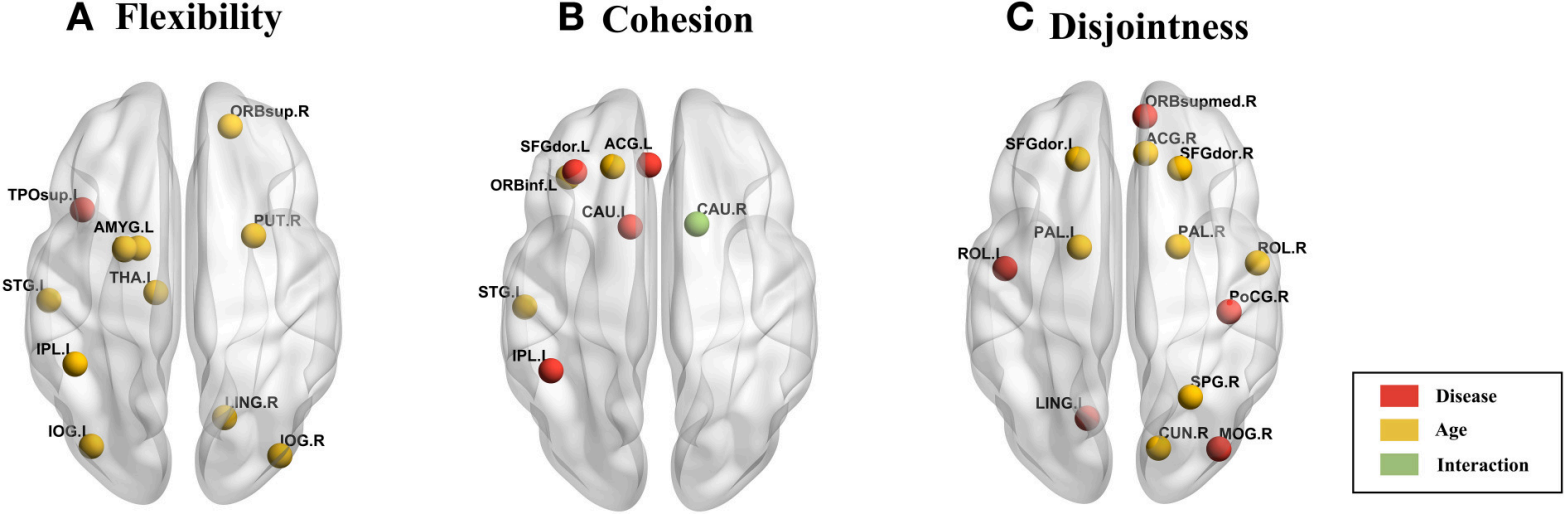
Brain plot of areas showing significant effect of age and disease on **(A)** flexibility, **(B)** cohesion strength, and **(C)** disjointness.

We used 2-factor ANOVA with factors as disease (2 levels: TD, ASD) and age (3 levels: children, adolescents and adults) to analyze their effects on regional flexibility, cohesion, and disjointness. We found a main effect of disease and of age on flexibility. The superior temporal gyrus (TPOsup) shows significantly [*F*_(1, 144)_ = 4.66, *p* = 0.03, partial eta-squared = 0.06 - medium effect size] reduced flexibility in ASD. To confirm that this difference was not driven by the difference in overall variance of the node but purely by the dynamic reconfiguration caused by the reduced flexibility, we also controlled for the mean dFCvar connections of this particular node. We still found a significant difference (*p* < 0.01) between ASD and TD groups. We also found several regions that show effect of age including: superior frontal orbital [ORBsup, *F*_(1, 144)_ = 3.92, *p* = 0.02], PAL [*F*_(1, 144)_ = 6.93, *p* = 0.001], amygdala (AMYG), cuneous (CUN), inferior occipital gyrus (IOG), left inferior parietal (IPL), angular gyrus (ANG), caudate nucleus (CAU), putamen (PUT), thalamus (THAL), SFGdor, and left superior temporal (STG). Comparisons on group statistics of pallidus gyrus (periphery region) showed a significant increase in flexibility in adults as compared to both adolescents (*p* = 0.01) and children (*p* = 0.0002) while the superior frontal orbital (periphery region) shows a significant (*p* = 0.005) increase of flexibility in adults as compared to adolescents (Table 3, Figure 6A).

**Table 3:** Areas showing significant effect (*p* < 0.05, FDR corrected) of age and disease on flexibility.

**Disease**					**Flexibility (SD)**		
**ROI**	**Name**	**Abbreviation**	**Network**	***p***	**ASD**	**TD**	
83	Temporal_Pole_Sup_L	TPOsup.L	Attention	0.036	0.092 (0.043)	0.107 (0.035)	
**Age**					**Children**	**Adolescents**	**Adults**
6	Frontal_Sup_Orb_R	ORBsup.R	DMN	0.021	0.100 (0.034)	0.095 (0.037)	0.118 (0.034)
41	Amygdala_L	AMYG.L	Subcortical	0.011	0.095 (0.040)	0.094 (0.036)	0.116 (0.037)
48	Lingual_R	LING.R	Visual	0.043	0.090 (0.029)	0.074 (0.034)	0.084 (0.036)
53	Occipital_Inf_L	IOG.L	Visual	0.008	0.104 (0.033)	0.084 (0.035)	0.103 (0.044)
54	Occipital_Inf_R	IOG.R	Visual	0.013	0.108 (0.034)	0.087 (0.037)	0.105 (0.031)
61	Parietal_Inf_L	IPL.L	Attention	0.039	0.102 (0.039)	0.091 (0.032)	0.108 (0.038)
74	Putamen_R	PUT.R	Subcortical	0.021	0.089 (0.043)	0.082 (0.037)	0.105 (0.038)
75	Pallidum_L	PAL.L	Subcortical	0.001	0.089 (0.038)	0.089 (0.038)	0.119 (0.044)
77	Thalamus_L	THA.L	Subcortical	0.030	0.085 (0.038)	0.091 (0.046)	0.110 (0.038)
81	Temporal_Sup_L	STG.L	Sensorimotor	0.011	0.078 (0.046)	0.075 (0.036)	0.102 (0.048)

On analyzing the effects of aging and disease on cohesion strength as well as disjointness for each individual node, we found certain nodes that show significant effect (*p* < 0.05) of disease, age, and their interaction. Specifically, we found that attention areas like middle frontal, inferior frontal orbital and the parietal node as well as the caudate nucleus show significant decrease in cohesion in ASD (*p* < 0.05, partial η^2^ ~ 0.05). While STG showed an increase in cohesion with age, areas like caudate nucleus, superior and inferior frontal areas show a general trend of decrease with age (Figure 6B, Table 4).

**Table 4:** Nodes showing significant effect (*p* < 0.05, FDR corrected) of age, disease, and interaction on cohesion strength.

**Effect of disease**					**Node cohesion strength (SD)**		
**ROI**	**Name**	**Abbreviation**	**Network**	***p***	**ASD**	**TD**	
7	Frontal_Mid_L	MFG.L	Attention	0.021	65.8 (10.4)	77.9 (22.6)	
15	Frontal_Inf_Orb_L	ORBinf.L	Attention	0.029	65.2 (9.91)	77.9 (22.8)	
31	Cingulum_Ant_L	ACG.L	DMN	0.014	55.3 (9.72)	69.4 (20.1)	
61	Parietal_Inf_L	IPL.L	Attention	0.017	75.4 (13.1)	64.8 (10.8)	
71	Caudate_L	CAU.L	Subcortical	0.016	62.9 (10.3)	72.9 (13.5)	
72	Caudate_R	CAU.R	Subcortical	0.012	62.2 (7.51)	74.3 (20.7)	
**Effect of age**					**Children**	**Adolescents**	**Adults**
3	Frontal_Sup_L	SFGdor.L	DMN	0.035	80.4 (19.4)	66.2 (13.4)	61.9 (12.6)
15	Frontal_Inf_Orb_L	ORBinf.L	Attention	0.023	74.5 (21.7)	79.2 (19.2)	58.1 (6.2)
72	Caudate_R	CAU.R	Subcortical	0.024	83.9 (27.5)	65.9 (14.7)	67.3 (10.5)
81	Temporal_Sup_L	STG.L	Sensorimotor	0.019	50.6 (13.9)	61.4 (16.6)	71.2 (16.5)
**Effect of Interaction**							
72	Caudate_R	CAU.R	Subcortical	0.004			

We found areas showing significant main effect of disease and age on node disjointness, but not of their interaction. The rolandic operculum gyrus, lingual and occipital gyrus as well as the medial frontal orbital show a significant increase in disjointness in ASD (*p* < 0.05, partial η^2^ ~ 0.06). Further, several nodes in DMN and Sensorimotor network show significant decrease in node disjointness with aging (*p* < 0.05, partial η^2^ ~ 0.07) (Figure 6C, Table 5). Overall, ASD shows an increase in disjointness and a decrease in node cohesion strength.

**Table 5:** Nodes showing significant effect (*p* < 0.05, FDR corrected) of age, disease, and interaction on node disjointness.

**Effect of disease**					**Node disjointness (SD)**		
**ROI**	**Name**	**Abbreviation**	**Network**	***p***	**ASD**	**TD**	
17	Rolandic_Oper_L	ROL.L	Sensorimotor	0.012	0.0039 (0.005)	0.0022 (0.004)	
26	Frontal_Med_Orb_R	ORBsupmed.R	DMN	0.039	0.0048 (0.007)	0.0034 (0.004)	
47	Lingual_L	LING.L	Visual	0.037	0.0047 (0.007)	0.0029 (0.006)	
53	Occipital_Inf_L	IOG.L	Visual	0.009	0.0074 (0.006)	0.0045 (0.001)	
58	Postcentral_R	PoCG.R	Sensorimotor	0.009	0.0067 (0.006)	0.0041 (0.006)	
**Effect of age**					**Children**	**Adolescents**	**Adults**
3	Frontal_Sup_L	SFGdor.L	DMN	0.014	0.0075 (0.006)	0.0040 (0.005)	0.0055 (0.006)
4	Frontal_Sup_R	SFGdor.R	DMN	0.037	0.0051 (0.003)	0.0061 (0.004)	0.0030 (0.006)
18	Rolandic_Oper_R	ROL.R	Sensorimotor	0.001	0.0019 (0.006)	0.0020 (0.007)	0.0052 (0.005)
32	Cingulum_Ant_R	ACG.R	DMN	0.028	0.0067 (0.005)	0.0063 (0.004)	0.0033 (0.006)
46	Cuneus_R	CUN.R	Visual	0.005	0.0032 (0.005)	0.0029 (0.006)	0.0060 (0.007)
60	Parietal_Sup_R	SPG.R	Sensorimotor	0.029	0.0063 (0.007)	0.0056 (0.006)	0.0027 (0.004)
75	Pallidum_L	PAL.L	Subcortical	0.031	0.0036 (0.005)	0.0054 (0.004)	0.0074 (0.006)
76	Pallidum_R	PAL.R	Subcortical	0.033	0.0066 (0.005)	0.0090 (0.006)	0.0060 (0.008)

### Correlation of Dynamic Metrics (Local Dynamics) With ADOS Scores

To analyze the relation between symptom severity and dynamic measures—flexibility, cohesion strength, and disjointness, we found their correlations with the ADOS scores. We did not find a significant correlation between mean whole brain flexibility scores with ADOS scores. At the functional network level, we found the mean flexibility score by averaging the individual scores of each node in a functional network. We found that an increase in flexibility score of visual system areas positively correlated with the ADOS total symptom severity score (*r* = 0.37, *p* < 0.01); ADOS Social score (*r* = 0.38, *p* < 0.01, uncorrected). We did not find any significant correlation with the other functional networks. At the level of individual ROIs, we found that the flexibility of several visual and auditory areas showed positive correlation with ADOS total and communication scores (Supplementary Table 5 and Supplementary Figure 1). Further, on dividing the subjects into 3 age groups - children, adolescents and adults, we found that adolescents and adults show a very strong positive correlation of ADOS social score and flexibility in rigid regions. Children show a strong negative correlation of ADOS score and flexibility of DMN region—which is a flexible periphery region (Table 6).

**Table 6:** Areas showing significant correlation between region flexibility and ADOS social scores in each age group.

**Node flexibility**					
**ROI**	**Abbreviation**	**Network**	**Name**	***r***	***p***
**CHILDREN**					
50	SOG.R	Visual	Occipital_Sup_R	0.50	0.012
86	MTG.R	DMN	Temporal_Mid_R	−0.41	0.037
**ADOLESCENTS**					
17	ROL.L	Sensorimotor	Rolandic_Oper_L	0.46	0.023
43	CAL.L	Visual	Calcarine_L	0.52	0.008
45	CUN.L	Visual	Cuneus_L	0.41	0.036
46	CUN.R	Visual	Cuneus_R	0.47	0.019
48	LING.R	Visual	Lingual_R	0.53	0.007
50	SOG.R	Visual	Occipital_Sup_R	0.49	0.010
72	CAU.R	Subcortical	Caudate_R	0.47	0.022
**ADULTS**					
21	OLF.L	Sensorimotor	Olfactory_L	0.69	0.004
43	CAL.L	Visual	Calcarine_L	0.61	0.012
45	CUN.L	Visual	Cuneus_L	0.56	0.027

On analyzing correlation between the dynamic properties of node disjointness as well as node cohesion strength with ADOS scores, we find that for adults, the mean node disjointness i.e., the average over the whole brain; shows a significant positive correlation (*r* = 0.45, *p* < 0.01) with the symptom severity ADOS scores. We also find several nodes that show significant correlation of cohesion strength with the ADOS scores. We have listed the details of the correlations with ADOS scores in Tables 7, 8.

**Table 7:** Nodes showing significant correlation of node cohesion strength with ADOS social score.

**ROI**	**Abbreviation**	**ROI name**	**Network**	***r***	***p***
**CHILDREN**					
7	MFG.L	Frontal_Mid_L	Attention	0.44	0.021
13	IFGtriang.L	Frontal_Inf_Tri_L	Attention	0.40	0.044
23	SFGmed.L	Frontal_Sup_Medial_L	DMN	0.47	0.009
**ADOLESCENT**					
45	CUN.L	Cuneus_L	Visual	0.41	0.035
**ADULT**					
18	ROL.R	Rolandic_Oper_R	Sensorimotor	−0.60	0.011
43	CAL.L	Calcarine_L	Visual	0.67	0.006
51	MOG.L	Occipital_Mid_L	Visual	0.56	0.033
57	PoCG.L	Postcentral_L	Sensorimotor	−0.52	0.038

**Table 8:** Nodes showing significant correlation of node disjointness with ADOS social score.

**ROI**	**Abbreviation**	**ROI name**	**Network**	***r***	***p***
**CHILDREN**					
14	IFGtriang.R	Frontal_Inf_Tri_R	Attention	0.46	0.022
34	DCG.R	Cingulum_Mid_R	Subcortical	0.57	0.003
84	TPOsup.R	Temporal_Pole_Sup_R	Sensorimotor	0.51	0.009
**ADOLESCENT**					
24	SFGmed.R	Frontal_Sup_Medial_R	DMN	−0.57	0.003
46	CUN.R	Cuneus_R	Visual	0.52	0.008
48	LING.R	Lingual_R	Visual	0.55	0.005
82	STG.R	Temporal_Sup_R	Sensorimotor	0.47	0.012
86	MTG.R	Temporal_Mid_R	DMN	−0.56	0.004
**ADULT**					
42	AMYG.R	Amygdala_R	Subcortical	0.52	0.039
50	SOG.R	Occipital_Sup_R	Visual	0.57	0.021

Overall, the cohesion strength of sensorimotor regions shows a negative correlation with ADOS scores for ASD adults while cohesion strength of visual, attention and DMN areas show positive correlation. DMN areas show negative correlation of disjointness with ADOS scores and vice versa for sensorimotor areas.

## Discussion

Autism is a neurodevelopmental disorder characterized by altered neural network dynamics. The presence of alterations in the dynamic configuration of the resting-state functional connectome and their association with ASD symptom severity are yet to be studied. To address this gap in knowledge, we applied temporal modularity metrics to analyze dynamics in resting-state fMRI data.

In our first analysis, we found that mean dFCvar between the Attention and DMN networks is positively correlated with the ADOS scores (Table 2). This indicates that the inter-subject variability is related to the symptom severity. It has been reported that higher dFCvar is associated with better performance in task and poor performance in resting-state (Douw et al., 2016). Similarly, Lin et al. (2016) reported that higher variability in the connection strength of posterior cingulate cortex (PCC) to other DMN areas in the resting-state is related to slower reaction times on a subsequent attention task. The hypervariance in ASD could lead to a globally disconnected state in its dynamics as reported in a previous study (Rashid et al., 2018). These results taken together indicate that there could be a relation between the atypical hypervariance in ASD which leads to increase in ADOS score and decrease in cognitive performance. We also found significant number of hyper-variable small, medium and long-range connections in three groups (Figure 2). The long-range connections define the backbone of the functional network and often connect the hubs regions to minimize wiring and energy costs (Chen et al., 2017). In ASD, the hypervariance in the long-range connections could cause instability in information transmission between hubs. Interestingly, for adolescents, we found higher number of hyper-variable short-range connections. The hypervariance in short-range connections could indicate instability of local-module connectivity (Chen et al., 2017). Further, several nodes including Frontal_Inf_Orb and Caudate showed both hyper-variability in connection strength and altered modular organization (flexibility) in ASD (Figures 2, 3).

In the next analysis, we explored dynamic network measures such as network flexibility, cohesion and disjointness to understand changes in functional connectivity that occur over time. Recent work in the field of network flexibility has centered around the relationship between task-performance or task-sentiment analysis with dynamic reconfiguration of the brain (Park et al., 2017; Telesford et al., 2017) especially in the memory areas (Douw et al., 2015). Changes in flexibility have been associated with mood (Betzel et al., 2017), inter-subject differences have been linked to learning (Bassett et al., 2011), working memory performance (Braun et al., 2015), and reinforcement learning (Gerraty et al., 2018). The metric has also been found to correlate with schizophrenia risk, and is altered by an NMDA-receptor antagonist (Braun et al., 2016). Flexibility also has age-based variation (Schlesinger et al., 2017). Recent work has established that there is a significant negative correlation between flexibility and modularity using task-based fMRI studies (Ramos-Nuñez et al., 2017).

In our study, we found that such a correlation exists even in the resting-state functional brain as well. We found that while dynamic flexibility correlates negatively with the static metric of modularity in TD, such a correlation does not exist in ASD (Figure 4). However, both TD and ASD showed a range of flexibilities, indicating that the whole-brain flexibility remains intact. This indicates that the modular re-organization with variations in flexibility is not observed in ASD. It is possible that there are network/nodal changes that compensate for the change in flexibility. We also found that modularity scores did not correlate with the symptom severity ADOS score while network-based flexibility scores showed a significant positive/negative correlation (Figure 5). This further indicates that static network measures are unable to capture the underlying variability in ASD with respect to the ADOS scores. In contrast, dynamic network measures have significant predictive power of ADOS scores.

We found that in the resting-state functional brain, regions with lower flexibility are those involved in visual, hearing and motor processes while those with higher flexibility are those typically associated with the default mode network, cognitive control and executive function. This organization has been previously reported in task-based fMRI studies (Cole et al., 2013; Braun et al., 2015; Mattar et al., 2015; Schlesinger et al., 2017). Cole et al. (2013) reported that higher flexibility in fronto-pareital network was associated with better task performance. de Lacy et al. (2017) reported that the number of state transitions were reduced in ASD as compared to TD, due to disruptions in the fronto-pareital network and impaired state transitions in the cingulo-opercular systems. In our study, we found that there is reduced flexibility of periphery regions (DMN, subcortical and attention areas) in ASD which could impair the state transitions.

Although network flexibility did not show disease or age effect, we found regions (nodes) that show their independent effect. Significant effect of disease is observed in TPOsup, which is an important area linked to verbal and non-verbal communication identified to show abnormal behavior in autism. It is a key periphery region which shows increased flexibility in the TD group. The decrease of flexibility of TPOsup could contribute to the nature of atypical verbal behavior observed in ASD. Only one region shows the effect of disease while several regions show correlation of ADOS score and flexibility. We hypothesize that in the ANOVA analysis, the underlying variability in ASD with respect to symptom severity is lost as they are considered as a single group. We also observed that ASD showed an atypical decrease in node cohesion strength and an increase in disjointness. Telesford et al. (2017) found that node cohesion strength showed positive correlation with performance in a task. Our results suggest that for resting-state dynamics, sensorimotor and visual regions show high cohesion strength while DMN shows relatively high disjointness.

Further, cohesion strength of sensorimotor regions correlates negatively (*r* = −0.6) with ADOS symptom severity score (Table 6), while regions of other networks show positive correlation (*r* ~ 0.5). The findings suggest that while sensorimotor areas are the least flexible, they are required to be less cohesive as well.

Our study differs in several aspects from previously reported dynamic whole-brain network-level investigations in ASD (Chen et al., 2017; de Lacy et al., 2017; Rashid et al., 2018). Most of the previous studies on dynamic functional connectivity in ASD combined sliding window analysis with *k*-means clustering to identify common brain states among all subjects (both ASD and TD). On the other hand, Watanabe and Rees (2017) concatenated the timeseries of ASD subjects to characterize the energy landscape of ASD. However, in our study, we have captured the variability among subjects by performing dynamic analysis on each individual subject. We uncovered the effect of development, disease, and their interactions on the dynamic metrics.

## Conclusion

To the best of our knowledge, this is one of the first studies to use dynamic modularity metrics like flexibility and cohesion to study altered dynamics in ASD and TD.

We found very high correlation of ADOS score with the flexibility and disjointness of Sensorimotor regions. This is indicative of the importance of maintaining the cohesion and rigidity of motor cortex regions in TD. It can be noted that in our study, static modularity does not show a correlation with ADOS scores. Using temporal modularity metrics for dynamic analysis is a recent approach and lacks standardization across clinical studies. Whole brain connectivity anomaly and differences are highly sensitive to the parameters used while partitioning the BOLD timeseries into instantaneous dynamic FC matrices. To study the robustness of our results, we also repeated the dFCvar analysis using different window parameters and found consistent results (Supplementary Information).

We also re-run the multi-layer modularity algorithm with different sets of parameter values for γ and ω. We found consistently a positive correlation between ADOS scores and visual network connections (Supplementary Information). Further, it is also extremely important to correct for head motion as it is an important confounding factor. Several studies have shown that not correcting for it can lead to spurious instantaneous connections, either increased or decreased. In our study, we have exercised scrutiny by using samples from a single site which are corrected for motion artifacts and considered samples with low framewise displacement scores that were approved by manual functional QA raters. This resulted in fewer samples in each age group affecting the statistics.

We observed very high correlation between dynamic connectivity metrics (flexibility, cohesion, and disjointness) and ADOS scores (Tables 6–8), however the *p*-values did not survive multiple comparisons. Increasing the number of participants in each group will help to validate our findings. Overall, this study provides insights into the patterns observed in the functional brain systems of TD and ASD from a dynamic perspective, which can be further extended using a longitudinal design, including a larger subject pool across sites and combining structural data as well.

## Ethics Statement

We used data from the ABIDE Preprocessed Initiative repository. Each site participating in ABIDE is required to confirm that the local Institutional Review Board (IRB) have allowed the collection as well as sharing of the data.

## Author Contributions

VH, DR, and PV have designed the research problem. VH, PV, RB, and DR carried out the original research. VH analyzed data. VH, PV, RB, and DR have all contributed in writing this original research article.

### Conflict of Interest Statement

The authors declare that the research was conducted in the absence of any commercial or financial relationships that could be construed as a potential conflict of interest.
