# Supplementary material for: Atypical Flexibility in Dynamic Functional Connectivity Quantifies the Severity in Autism Spectrum Disorder
Source: Front Hum Neurosci. 2019 Feb 1;13:6. doi: 10.3389/fnhum.2019.00006 (PMC6367662; doi:10.3389/fnhum.2019.00006)
Supplement: Supplementary file 1 [file Data_Sheet_1.PDF]

Supplementary Material

Atypical flexibility in dynamic functional connectivity quantifies the severity in autism spectrum disorder

Vatika Harlalka[1], Raju S. Bapi [2,3], P.K. Vinod [1*] and Dipanjan Roy[4*]

*** Correspondence:**
Dr. Dipanjan Roy

dipanjan.nbrc@gov.in

Dr. P.K. Vinod

vinod.pk@iiit.ac.in

1. Supplementary Information

1.1 Correlation calculation:

We used the non-parametric permutation method for calculating correlations in the dFCvar matrix between functional networks and ADOS scores. For each connection in dFCvar, correlation coefficients were calculated between the dFCvar and ADOS total score. We summed the number of significantly correlated ($p < 0.05$) connections. The subjects were shuffled to create a random pool, then the correlation coefficients between the dFCvar of each connection and ADOS total score were calculated in this shuffled dataset. The above step was repeated 5000 times and 5000 significantly positively correlated connections at random were obtained. Final *p*-value was calculated as the times in which the random sum of correlated connections were more than the actual sum of correlated connections divided by 5000.

1.2 Robustness with window parameters:

To analyze the robustness of our analysis with window parameters, we repeated the dynamic FC analysis with a window size of 40s and step size of 2. We found that for children, adolescents and adults, ASD showed significantly greater variability ($p < 0.05$) as compared to TD. For children, the cluster size is 44 connections, with majority connections (19 connections) being long range. For adolescents, the cluster size is 34 connections, with majority as short range (15 connections). For adults, the cluster size is 52 connections with 19 long range and 20 middle range connections. On correlating dFCvar with ADOS scores, we found that the DMN-DMN as well as DMN-Attention mean dFCvar showed significant positive correlation ($r > 0.3$, $p < 0.05$). Further, 8 connections were

identified that showed high correlation with ADOS score ($r > 0.5$, $p < 0.05$). Overall, the distribution of the hypervariant connections as well as ADOS correlation is consistent with our reported results.

1.3 Robustness with modularity maximization parameters:

An important issue is to select the topological scale of interest, which is focusing on a subset of gamma values. At the topological scale considered (gamma=1) in our study, a convergence to similar community structure over multiple runs was observed. We further demonstrate the robustness of our result by slightly varying the parameters from selected values as suggested by Betzel and Bassett (2017). We found consistently a significant positive correlation between flexibility scores of visual areas and ADOS scores in parameter range between 0.95-1.05 for gamma and omega values. Further, we also performed analysis in other topological scales (gamma=0.5 and gamma=1.5) and observed consistent results within other topological scales.

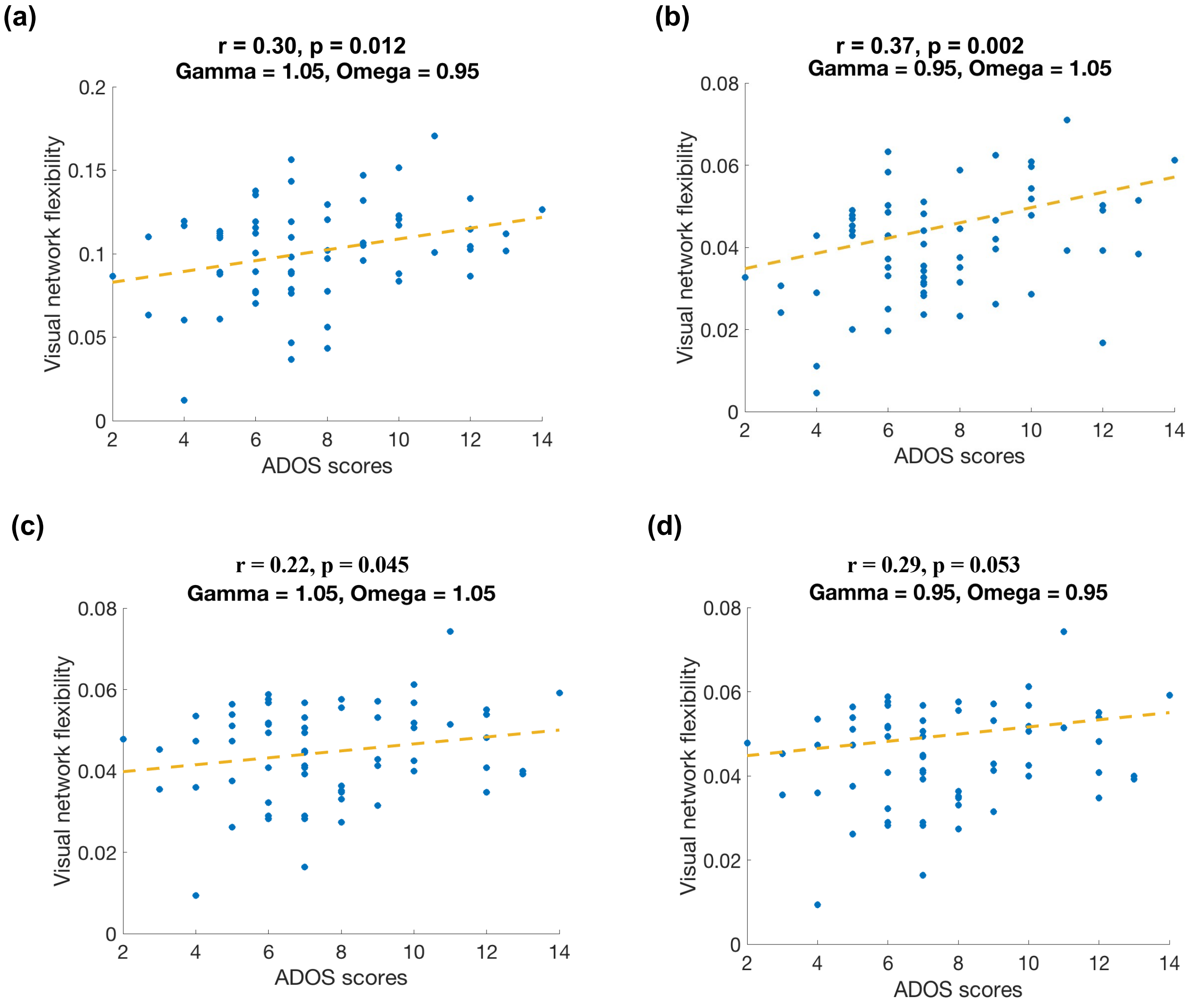

Other topological scales: varying gamma with fixed omega (= 1.0)

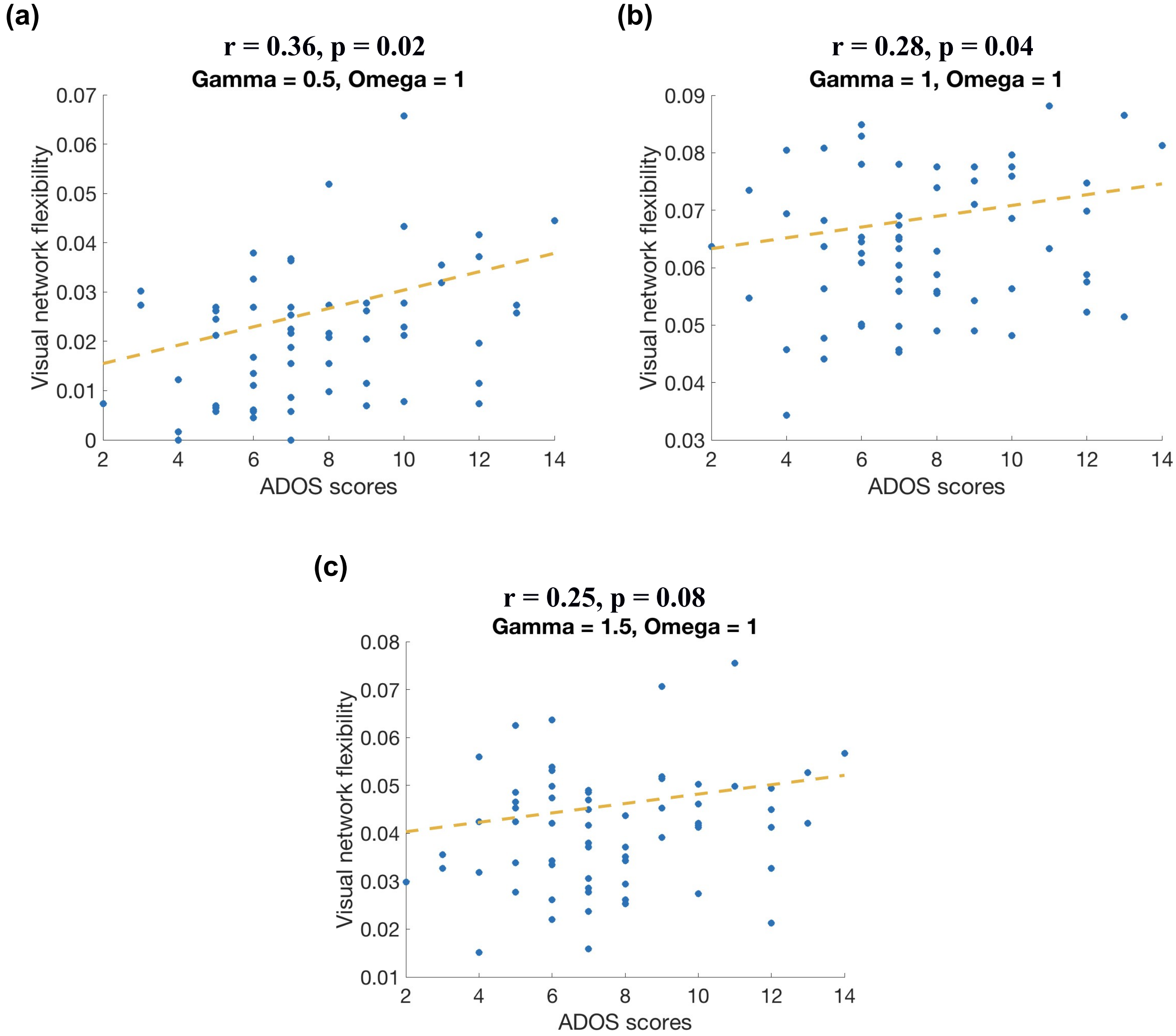

2. Supplementary Figures and Tables

2.1. Supplementary Figures

Supplementary Figure 1. Areas showing significant ($p < 0.05$) correlation of flexibility score with ADOS scores.

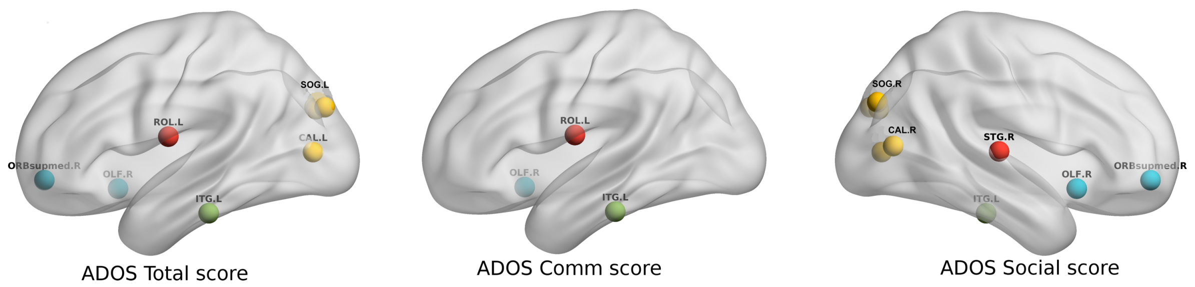

2.2. Supplementary Tables

Supplementary Table 1: Mapping between ROI names and abbreviations in AAL 90 ROI parcellation.

ROI	Regions	Abbreviation	ROI	Regions	Abbreviation	ROI	Regions	Abbreviation
1	Precentral_L	PreCG.L	31	Cingulum_Ant_L	ACG.L	61	Parietal_Inf_L	IPL.L
2	Precentral_R	PreCG.R	32	Cingulum_Ant_R	ACG.R	62	Parietal_Inf_R	IPL.R
3	Frontal_Sup_L	SFGdor.L	33	Cingulum_Mid_L	DCG.L	63	SupraMarginal_L	SMG.L
4	Frontal_Sup_R	SFGdor.R	34	Cingulum_Mid_R	DCG.R	64	SupraMarginal_R	SMG.R
5	Frontal_Sup_Orb_L	ORBsup.L	35	Cingulum_Post_L	PCG.L	65	Angular_L	ANG.L
6	Frontal_Sup_Orb_R	ORBsup.R	36	Cingulum_Post_R	PCG.R	66	Angular_R	ANG.R
7	Frontal_Mid_L	MFG.L	37	Hippocampus_L	HIP.L	67	Precuneus_L	PCUN.L
8	Frontal_Mid_R	MFG.R	38	Hippocampus_R	HIP.R	68	Precuneus_R	PCUN.R
9	Frontal_Mid_Orb_L	ORBmid.L	39	ParaHippocampal_L	PHG.L	69	Paracentral_Lobule_L	PCL.L
10	Frontal_Mid_Orb_R	ORBmid.R	40	ParaHippocampal_R	PHG.R	70	Paracentral_Lobule_R	PCL.R
11	Frontal_Inf_Oper_L	IFGoperc.L	41	Amygdala_L	AMYG.L	71	Caudate_L	CAU.L
12	Frontal_Inf_Oper_R	IFGoperc.R	42	Amygdala_R	AMYG.R	72	Caudate_R	CAU.R
13	Frontal_Inf_Tri_L	IFGtriang.L	43	Calcarine_L	CAL.L	73	Putamen_L	PUT.L
14	Frontal_Inf_Tri_R	IFGtriang.R	44	Calcarine_R	CAL.R	74	Putamen_R	PUT.R
15	Frontal_Inf_Orb_L	ORBinf.L	45	Cuneus_L	CUN.L	75	Pallidum_L	PAL.L
16	Frontal_Inf_Orb_R	ORBinf.R	46	Cuneus_R	CUN.R	76	Pallidum_R	PAL.R
17	Rolandic_Oper_L	ROL.L	47	Lingual_L	LING.L	77	Thalamus_L	THA.L
18	Rolandic_Oper_R	ROL.R	48	Lingual_R	LING.R	78	Thalamus_R	THA.R
19	Supp_Motor_Area_L	SMA.L	49	Occipital_Sup_L	SOG.L	79	Heschl_L	HES.L
20	Supp_Motor_Area_R	SMA.R	50	Occipital_Sup_R	SOG.R	80	Heschl_R	HES.R
21	Olfactory_L	OLF.L	51	Occipital_Mid_L	MOG.L	81	Temporal_Sup_L	STG.L
22	Olfactory_R	OLF.R	52	Occipital_Mid_R	MOG.R	82	Temporal_Sup_R	STG.R
23	Frontal_Sup_Medial_L	SFGmed.L	53	Occipital_Inf_L	IOG.L	83	Temporal_Pole_Sup_L	TPOsup.L
24	Frontal_Sup_Medial_R	SFGmed.R	54	Occipital_Inf_R	IOG.R	84	Temporal_Pole_Sup_R	TPOsup.R
25	Frontal_Mid_Orb_L	ORBsupmed.L	55	Fusiform_L	FFG.L	85	Temporal_Mid_L	MTG.L
26	Frontal_Mid_Orb_R	ORBsupmed.R	56	Fusiform_R	FFG.R	86	Temporal_Mid_R	MTG.R
27	Rectus_L	REC.L	57	Postcentral_L	PoCG.L	87	Temporal_Pole_Mid_L	TPOmid.L
28	Rectus_R	REC.R	58	Postcentral_R	PoCG.R	88	Temporal_Pole_Mid_R	TPOmid.R
29	Insula_L	INS.L	59	Parietal_Sup_L	SPG.L	89	Temporal_Inf_L	ITG.L
30	Insula_R	INS.R	60	Parietal_Sup_R	SPG.R	90	Temporal_Inf_R	ITG.R

Supplementary Table 2. Between-networks and within-network correlation of dFCvar with ADOS scores.

**Network_1**	**Network_2**	**correlation**	**p**
DMN	DMN	0.34	0.04
	visual	-0.05	0.66
	attention	0.39	0.01
	subcortical	0.15	0.22
	sensorimotor	0.16	0.2
Visual	visual	0.22	0.07
	attention	0.06	0.58
	subcortical	0.05	0.67
	sensorimotor	0.17	0.15
attention	attention	0.2	0.1
	subcortical	0.17	0.16
	sensorimotor	0.19	0.12
subcortical	subcortical	0.24	0.051
	sensorimotor	0.24	0.052
sensorimotor	sensorimotor	0.23	0.06

Supplementary Table 3. Difference in flexibility measure between networks.

Network 1	Network 2	Difference	Std error	p-Value	95% CI-lower	95% CI-upper
DMN	Sensorimotor	0.00866	0.00209	0.00034	0.00294	0.01437
DMN	Visual	0.00726	0.00232	0.01495	0.00094	0.01358
Sensorimotor	Subcortical	-0.01177	0.00166	0.00E+00	-0.0163	-0.00725
Sensorimotor	Attention	-0.01087	0.00204	0.00E+00	-0.01645	-0.0053
Visual	Subcortical	-0.01038	0.00229	6.00E-05	-0.01663	-0.00413
Visual	Attention	-0.00948	0.00221	0.00017	-0.0155	-0.00346

Supplementary Table 4. Group statistics of flexibility score of each network.

Network	Mean	Std dev
Default Mode	0.096	0.023
Sensorimotor	0.087	0.027
Visual	0.089	0.027
Subcortical	0.099	0.024
Attention	0.098	0.022

Supplementary Table 5. Areas showing significant correlation of regional flexibility score with ADOS score (* indicates that p-value survived FDR correction).

ROI #	Name	Network	Abbreviation	r	p
ADOS COMMUNICATION score					
17	Rolandic_Oper_L	Sensorimotor	ROL.L	0.31	0.012
22	Olfactory_R	Sensorimotor	OLF.R	0.25	0.041
89	Temporal_Inf_L	Attention	ITG.L	0.29	0.017
ADOS SOCIAL score					
22	Olfactory_R	Sensorimoto r	OLF.R	0.32	0.01
26	Frontal_Med_Orb_ R	DMN	ORBsupmed. R	0.28	0.02
43	Calcarine_L	Visual	CAL.L	0.35	0.0038
44	Calcarine_R	Visual	CAL.R	0.26	0.037
45	Cuneus_L	Visual	CUN.L	0.29	0.018
49	Occipital_Sup_L	Visual	SOG.L	0.28	0.021
50	Occipital_Sup_R	Visual	SOG.R	0.45	0.00014*
82	Temporal_Sup_R	Sensorimotor	STG.R	0.29	0.049
89	Temporal_Inf_L	Attention	ITG.L	0.27	0.027
ADOS TOTAL score					
17	Rolandic_Oper_L	Sensorimotor	ROL.L	0.27	0.026
22	Olfactory_R	Sensorimotor	OLF.R	0.33	0.0075
26	Frontal_Med_Orb_ R	DMN	ORBsupmed.R	0.26	0.038
43	Calcarine_L	Visual	CAL.L	0.32	0.0096
45	Cuneus_L	Visual	CUN.L	0.29	0.018

46	Cuneus_R	Visual	CUN.R	0.25	0.045
49	Occipital_Sup_L	Visual	SOG.L	0.25	0.046
50	Occipital_Sup_R	Visual	SOG.R	0.42	0.00043*
89	Temporal_Inf_L	Attention	ITG.L	0.31	0.011
